# Physiological Acclimatization of the Liver to 180-Day Isolation and the Mars Solar Day

**DOI:** 10.1155/2020/2796510

**Published:** 2020-03-21

**Authors:** Hailong Chen, Ke Lv, Guohua Ji, Yanhong Yuan, Liang Lu, Fengji Liang, Kai Li, Zi Xu, Jianghui Xiong, Lina Qu, Yinghui Li

**Affiliations:** ^1^State Key Laboratory of Space Medicine Fundamentals and Application, China Astronaut Research and Training Center, 26 Beiqing Road, Haidian District, Beijing, China; ^2^Department of health technology research and development, SPACEnter Space Science and Technology Institute (Shenzhen), 4 Shamiao Road, Pingdi Street, Longgang District, Shenzhen 518117, China

## Abstract

Physiological changes in humans are evident under environmental conditions similar to those on a Mars mission involving both a space factor (long-term isolation) and a time factor (the Mars solar day). However, very few studies have investigated the response of the liver to those conditions. Serum protein levels, bilirubin levels, aminotransferase activities, blood alkaline phosphatase, gamma-glutamyltransferase, lipid levels, and serum cytokines interleukin-6 and interferon-*γ* levels were analyzed 30 days before the mock mission; on days 2, 30, 60, 75, 90, 105, 120, 150, and 175 of the mission; and 30 days after the mission, in four subjects in 4-person 180-day Controlled Ecological Life Support System Experiment. Serum protein levels (total protein and globulin) decreased and bilirubin increased under the isolation environment from day 2 and exhibited chronic acclimatization from days 30 to 175. Effects of the Mars solar day were evident on day 75. Blood lipid levels were somewhat affected. No obvious peak in any enzyme level was detected during the mission. The change tendency of these results indicated that future studies should explore whether protein parameters especially globulin could serve as indicators of immunological function exposure to the stress of a Mars mission.

## 1. Introduction

During a future manned mission to Mars, long-term isolation [1] and the Mars solar day [2] will be major stressors of astronauts. Long-term isolation impairs sleep, mood, and alertness [3]; compromises muscle strength [4]; induces changes in stress hormone levels and glucose metabolism [5, 6]; weakens the extent of healthy qi; triggers a free-fall in “Gan” (liver) failure; and disrupts the relationship between “Gan” and “Wei” (the stomach) [7]. The human circadian system evolved to synchronize strictly to the 24 h Earth day, but must entrain to the Mars solar day, almost 40 min longer than the Earth day, during a Mars mission [2]. Failure to entrain to the Mars solar day over several days will disrupt sleep; compromise endocrine physiology; impair cognition, alertness, mood, and vigilance; and predispose to poor performance [8–11]. The liver, a complex multifunctional organ, maintains bodily homeostasis [12], regulates substance and energy metabolism [13], and plays a front-line role in removing microbial components and toxins from portal blood [14]. However, the response of protein as well as bilirubin to long-term isolation, and the Mars solar day has not been characterized. To investigate changes in human liver function under such conditions, we evaluated human liver function markers in four subjects in the Controlled Ecological Life Support System (CELSS) experiment, which commenced in June 2016 to explore physiological, psychological, and behavioral changes during long-term isolation and exposure to the Mars solar day [15].

## 2. Materials and Methods

### 2.1. Subjects and Experiments

Four subjects (three males and one female, aged 29–43 years) participated in 4-person 180-day CELSS Experiment at the SPACEnter Space Science and Technology Institute (Shenzhen, China) from June to December 2016; this featured 180 days of isolation and a Mars solar day from 10 : 30 PM on day 71 to 10 : 30 PM on day 108. The CELSS featured six cabins. A cabin for crew and life support system facilitated daily work, cooking, and sleeping, and a cabin for resource system was used to dispose of nonedible plant materials and to produce CO_2_, and four Greenhouses used to cultivate 25 kinds of plants including wheat, potatoes, sweet potatoes, soybeans, peanuts, lettuce, cabbage, edible amaranth, cherry radish, tomatoes, and strawberries. The indoor air of each cabin was usually not ventilated, but was when the oxygen ratio became lower than 19.0% or the CO_2_ level lower than 500 ppm. The subjects and experimental environment have been described in detail elsewhere [15, 16].

### 2.2. Sample Collection and Storage

**Figure 1:**
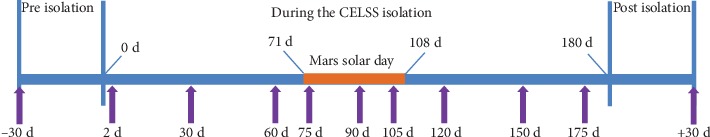
Experiment timeline and serum collecting time points.

Serum were collected from the four (fasting) subjects 30 days before the mission (−30d); on days 2, 30, 60, 75, 90, 105, 120, 150, and 175 during the mission (2d, 30d, 60d, 75d, 90d, 105d, 120d, 150d, 175d); and 30 days after the mission (+30d) (Figure 1). There was no smoking or alcohol consumption in the four subjects. Food, physical activity, temperature, and wakefulness cycle were all controlled well before each blood collection. All samples were stored at −80°C prior to analysis.

### 2.3. Measurement of Proteins, Enzymes, Bilirubins, and Lipids

The serum levels of total protein (TP), albumin (ALB), and globulin (GLB); the activities of alanine aminotransferase (ALT), aspartate aminotransferase (AST), alkaline phosphatase (ALP), and gamma-glutamyltransferase (GGT); and the levels of bilirubin (BIL), total bilirubin (TBIL), direct bilirubin (DBIL), indirect bilirubin (IBIL), and total triglycerides (TG) were measured. The levels of total cholesterol (TC), high-density lipoprotein cholesterol (HDL-C), and low-density lipoprotein cholesterol (LDL-C) were analyzed via the hexokinase method using a Hitachi 7600 Autoanalyzer (Hitachi Ltd., Tokyo, Japan).

### 2.4. Detection of Serum IL-6 and IFN-*γ*

The serum level of interleukin-6 (IL-6) was detected with enzyme-linked immunosorbent assay (ELISA) Kit (R&D, D6050, USA) and that of interferon-*γ* (IFN-*γ*) was detected with ELISA Kit (R&D, DIF50, USA).

### 2.5. Data Analysis

Data are presented as mean ± SEM. The small sample (four subjects) with mixed gender (three males and one female) limited the utility of statistical tests, so we only use average value to locate the difference in those time points and provide descriptive statistics and descriptive analyses.

## 3. Results

### 3.1. Protein Levels

**Figure 2:**
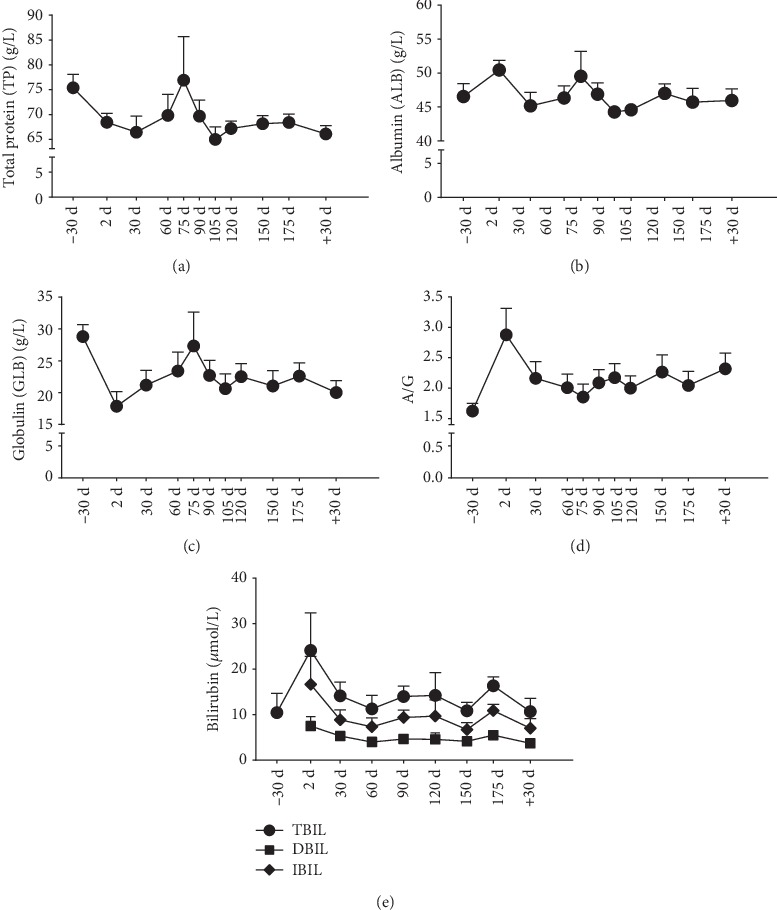
Protein and bilirubin levels. (a) TP; (b) ALB; (c) GLB; (d) A/G; (e) TBIL, DBIL, and IBIL; Data are expressed as means ± SEM.

The tendency of average total protein (TP) and globulin (GLB) levels was lower from 2d to 60d (minimum value was 88% or 62% of that on −30d, respectively), recovered to −30d levels by 75d, and lower again from 90d onwards (86% or 70% of that on −30d, respectively). However, the ALB level remained constant before, during, and after the mission, except for brief rises on 2d (1.1 fold of that on −30d) and 75d (1.1 fold of that on -30d). Therefore, the changes in the ALB to GLB (A/G) ratios showed exactly the opposite tendency in contrast with the observed results in the TP and GLB levels: the ratio was very high on 2d (1.8 fold of that on −30d), relatively lower on 75d, and relatively higher from 90d onwards (1.3 fold of that on −30d) (Figures 2(a)–2(d)).

### 3.2. Bilirubin Levels

The tendency of average total bilirubin (TBIL) level increased on 2d (to 2.3-fold −30d level) and 175d (to 1.6-fold −30d level). The direct bilirubin (DBIL) and indirect bilirubin (IBIL) levels exhibited similar changes (Figure 2(e)).

### 3.3. Serum Aminotransferase Activities

**Figure 3:**
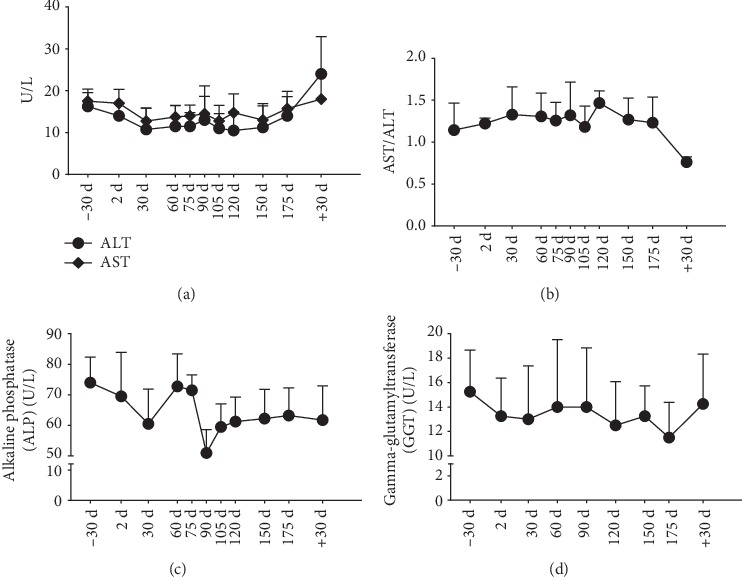
Enzymes. (a) ALT and AST; (b) AST/ALT; (c) ALP; (d) GGT. Data are expressed as means ± SEM. Presentation of results for ALT and AST with 8 time points has been permitted by *Frontiers in Physiology*.

Tendency of alanine aminotransferase (ALT) activity was decreased from 105d to 150d (minimum value was 65% of that on −30d), but was reactivated on +30d (to 1.5-fold that on −30d). Similarly, aspartate aminotransferase (AST) activity was reduced on 30d, 105d, and 150d during the mission (minimum value was 73% of that on −30d), but then rose to the level of −30d. The AST to ALT (AST/ALT) ratio did not change during or after the mission (Figures 3(a) and 3(b)).

### 3.4. Serum ALP and GGT Activities

Similar to the AST activity, the alkaline phosphatase (ALP) activity fell on 30d and after 90d; the minimum level was 68.9% that on −30d. Gamma-glutamyltransferase (GGT) activity was also low on 30d, 120d, and 175d (minimum value was 75% of that on −30d) (Figures 3(c) and 3(d)).

### 3.5. Blood Lipid Levels

**Figure 4:**
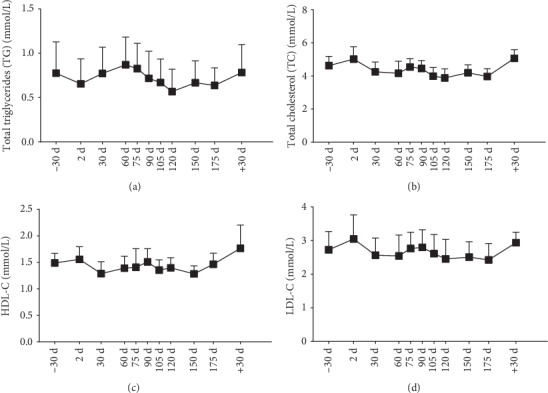
Blood lipid levels. (a) TG; (b) TC; (c) HDL-C; (d) LDL-C. Data are expressed as means ± SEM. Presentation of results for lipids with 8 time points has been permitted by *Frontiers in Physiology*.

Except for a low total triglycerides (TG) concentration on 120d (86% of that on −30d) and high-density lipoprotein cholesterol (HDL-C) level on +30d (1.2 fold of that on −30d), we found no change in blood lipid levels during or after the mission (Figure 4).

### 3.6. Serum IL-6 and IFN-*γ* Levels

**Figure 5:**
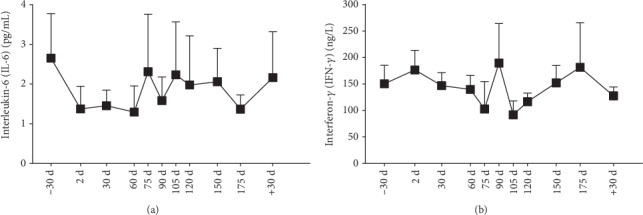
Serum IL-6 and IFN-*γ* levels. (a) IL-6; (b) IFN-*γ*. Data are expressed as means ± SEM.

Both of them decreased from 2d to 60d (minimum value was 49% or 93% of that on −30d, respectively), recovered to −30d levels by 75d (for interleukin-6 (IL-6)) or by 90d (for interferon-*γ* (IFN-*γ*)), and decreased again after 90d (60% of that on −30d for IL-6) or on 105d (61% of that on −30d for IFN-*γ*) (Figure 5).

## 4. Discussion

We found that both protein and bilirubin level changes under long-term (180-day) isolation might reflect the probable adaptation to isolation. Initial effects were followed by a recovery toward baseline; this persisted for a short time, followed by chronic acclimatization to a new steady state. We found decreases in TP and GLB levels, but increases in ALB and BIL levels and the A/G ratio, on day 2; blood homeostasis, in terms of both protein and bilirubin levels, was changed. These functions then rapidly recovered. However, long-term isolation (from day 30 to 175) was associated with new steady-state levels (between the normal and initial levels). Similar effects were evident for blood lipids (TG, TC, HDL-C, or LDL-C). The observed “initial effects–recovery–chronic acclimatization” process is similar to the effect of gravity on circadian timing [17].

The intrinsic human circadian period averages 24.2 h [18, 19], about 0.45 h less than the Mars solar day (24.67 h). Protein levels such as TP, ALB, and GLB rose and triggered effects exposure to the Mars solar day. However, the human circadian period can entrain to the Mars solar day, avoiding disturbance of the circadian rhythm, which can impair sleep, alertness, mood, and cognitive function [8, 9, 20]. We used fluorescent lamps in the six cabins and LED lamps [15, 16] to assist entrainment to the Mars solar day, and the effects on protein levels disappeared soon after day 75 of the mission.

A reduction in GLB level and an increase in the A/G ratio are indicators of poor immunological status and high susceptibility to infection. As a decrease in GLB level and a high A/G ratio were observed during the mission, the immunological profile may diverge from that characteristic of life on Earth. A prior report found a decrease in leukocyte numbers and increases in lymphocyte and monocyte numbers during 131 days of isolation [21].

To verify the relationship between GLB change and immunological function, we detected the serum IL-6 and IFN-*γ* levels and found that IL-6 change was consistent with the change of GLB. It was reported that IL-6 could promote GLB production and in particular regulate immunoglobulin synthesis through facilitating T-follicular helper-cell differentiation and IL-21 production [22, 23]. In addition, ALB could be used to predict the prognosis of cancer patients. Patients with high A/G ratio have better survival than patients with low A/G ratio [23]. Moreover, as both proinflammatory and anti-inflammatory cytokine, IL-6 might probably predominantly present anti-inflammatory effect opposite to that of IFN-*γ*. This intriguing phenomenon needs to be further studied.

Hepatocyte enzymes such as ALT and AST are released into the bloodstream when hepatocytes are injured or cell membrane integrity is compromised. Therefore, high serum ALT or AST activity levels reflect liver damage [24]. The serum activities of ALT, AST, ALP, and GGT did not change during the mission; thus, neither 180-day isolation nor the Mars solar day caused liver damage. Unexpectedly, ALT was activated on day +30, possibly associated with a burdensome postmission task.

A recent short-term study found no obvious changes in neurophysiological, neuropsychological, or cognitive function during 30 days of isolation [25]. However, this does not indicate that behavioral, psychological, physiological, and/or biochemical changes will not develop during long-term isolation (>100 days). Further research is needed.

Yuan et al. [26] showed that body weight decreased (mainly lean mass) on the 6th month during the isolation environment. We found that concentration of TP as well as GLB also reduced on 175^th^ day. This suggested that the change of protein level was in accordance with that of lean mass, which may be multiple factors including isolation, diet, workload, and training. However, there may be no relationship between lipid change and fat mass change, because fat mass was without clear trends under isolation station, as shown in the results of Yuan et al.

A limitation of the study is that we included only four subjects. We thus lack data of proteins, bilirubins, and lipids on a large sample and only give a descriptive analysis for the change tendency instead of statistical analysis. Additionally, we did not measure BIL concentration or GGT activity on day 75 of the mission, as the samples were lost. Another limitation is that we did not investigate the daily rhythmic changes of the proteins, bilirubins, enzymes, and lipids during Earth solar day and Mars solar day.

In summary, this study provides useful insights into how the protein and bilirubin react to long-term isolation and the Mars solar day. Our findings will contribute to planning a manned Mars mission. Future studies are required to clarify whether protein and bilirubin parameters especially GLB can serve as indicators of immunological function changes during a prolonged Mars mission.

## Data Availability

All data are available.
